# Texas to Be Congratulated

**Published:** 1897-12

**Authors:** 


### Texas to Be Congratulated.

Now that the epidemic of yellow fever is at an end in all parts of the South, it is time for Texas to call the whole country's attention to this State's wonderful success in preventing the dreaded disease from touching within its borders.

Within 350 miles of a city that was affected with over 1800 cases, and with the disease an epidemic in a border State for over three months, Texas did not develop more than a dozen "suspicious" fever patients, and not [but] one death. Even the few cases that were called yellow fever have been disputed by prominent physicians, and the fact that the disease did not spread would certainly lend countenance to the belief that not one genuine case of the scourge has been seen within the confines of this State.

The immunity of Texas from the disease is due to the prompt and efficient quarantine system put into effect by the State and by such cities as Houston and Galveston. State Health Officer Swearingen deserves much credit for his constant watchfulness, earnest efforts and his stubborn stand for precautionary methods, against all opposition. The mayors and boards of health of Galveston and Houston, as well as the officials of the border towns, are also to be congratulated upon their work of quarantine inspection, as well as upon their enforcement of sanitary regulations. A strict quarantine system caused much hardship and interfered with business to a great extent in this State, but it saved Texas from an epidemic that would have been a thousand times worse.

It has now been demonstrated that Texas can and will prevent the yellow fever from finding a resting place within its wide limits, and this should be blazoned forth to the world. When a few suspicious cases of fever were discovered at Houston and Galveston, many of the Northern and Eastern papers announced in startling press dispatches that the scourge was raging in Texas. Now let the people and the press of Texas unite in calling attention to the encouraging fact that this State succeeded in preventing such a visitation. Let it be known to the intending immigrants everywhere that Texas is able to protect them in every way; that not only is her soil inviting, her laws the workingman's safeguard, her sources of wealth innumerable, but that her officers—State, county and municipal—are prepared to place around them all the safeguards of health.—*Houston Post.*

